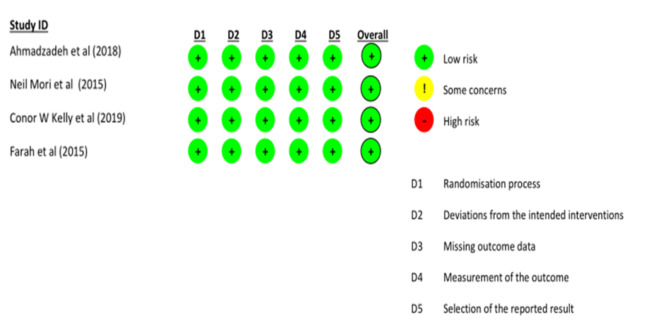# Association of white blood cell parameters with metabolic syndrome: A systematic review and meta-analysis of 168,000 patients

**DOI:** 10.1097/MD.0000000000037331

**Published:** 2024-03-08

**Authors:** Aysal Mahmood, Hoorain Haider, Saba Samad, Danisha Kumar, Aimen Perwaiz, Rabeea Mushtaq, Abraish Ali, Muhammad Zain Farooq, Hadi Farhat

**Affiliations:** a Dow University of Health Sciences (DUHS), Karachi, Pakistan; b Moffitt Cancer Center, University of South Florida, Tampa, FL; c Faculty of Medicine, Lebanese University, Beirut, Lebanon.

**Keywords:** leukocyte, meta-analysis, metabolic syndrome, neutrophil, white blood cell

## Abstract

**Background:**

Leukocyte parameters are predicted to be affected in patients with metabolic syndrome (MetS). We conducted a systematic review and meta-analysis to study the association between white blood cell parameters (WBC) in people with and without MetS.

**Methods:**

PubMed, EMBASE, Scopus and Cochrane Library databases were searched according to the study protocol. The standardized mean difference (SMD) and 95% confidence intervals (CI) of leukocyte markers between individuals with and without MetS were pooled using an inverse variance model. Additionally, a subgroup analysis by sex was performed where possible. Methodological quality assessment was conducted using the Newcastle-Ottawa scale (NOS) for observational studies and the Cochrane Risk of Bias tool 2.0 for Randomized Controlled Trials (RCTs).

**Results:**

Of 6068 articles identified, 63 were eligible for the study. Compared to controls, individuals with MetS showed significantly higher concentrations of total leukocyte count (SMD [95% CI]: 0.60 [0.55–0.65]; *P* < .00001; *I*^2^ = 100%), neutrophil counts (0.32 [0.28–0.37]; *P* < .00001; *I*^2^ = 99%), lymphocyte counts (0.15 [0.07–0.23]; *P* = .0004; *I*^2^ = 100%), basophil counts (0.01 [0.00–0.02]; *P* = .02; *I*^2^ = 98%), monocyte counts (0.05 [0.02–0.09]; *P* = .003; *I*^2^ = 99%), and neutrophil-to-lymphocyte ratio (0.24 [0.15–0.33]; *P* < .00001; *I*^2^ = 98%). There were no significant differences in the eosinophil count (0.02 [−0.01 to 0.05]; *P* = .19; *I*^2^ = 96%) and monocyte-to-lymphocyte ratio (0.06 [−0.05 to 0.17]; *P* = .27; *I*^2^ = 100%) between patients with and without MetS, however, the lymphocyte-to-monocyte ratio (0.52 [−0.81 to −0.23]; *P* = .0005; *I*^2^ = 52%) tended to be significantly lower in patients with MetS.

**Conclusion:**

Biomarkers such as total leukocyte count, neutrophil count, lymphocyte count, basophil count, monocyte count and neutrophil-to-lymphocyte ratio are associated with higher levels in patients in MetS and thus can potentially be used for early detection of MetS.

## 1. Introduction

The American Heart Association and the National Heart, Lung, and Blood Institute define metabolic syndrome as a cluster of correlated metabolic risk factors that predispose an individual to the development of atherosclerotic cardiovascular disease.^[1]^ Several panels of specialists in the field have proposed standard diagnostic criteria to help investigate these risk factors in a clinical setting. Though there are certain differences in the proposed criteria by various authorities, it is generally agreed upon that the main culprits in the development of metabolic syndrome (MetS) are the presence of atherogenic dyslipidemias, i.e., increased low-density lipoprotein and high-density lipoprotein (HDL), elevated blood pressure, hyperglycemia along with visceral adiposity. Thus, the International Diabetes Federation proposes MetS as the presence of central obesity (defined as waist circumference but can be assumed if body mass index > 30 kg/m^2^) with ethnicity-specific values, plus 2 of the following: Systolic blood pressure ≥ 130 mm Hg or Diastolic ≥ 85 mm Hg, Triglycerides ≥ 150 mg/dL (1.7 mmol/L), HDL < 40 mg/dL (men) (1.03 mmol/L) or < 50 mg/dL (1.29 mmol/L) (women), fasting blood sugar ≥100 mg/dL (5.6 mmol/L) or diagnosed type 2 diabetes mellitus (T2DM).^[2]^

Metabolic risk factors, such as diabetes, hypertension, and hyperlipidemia, play a significant role in the development of cardiovascular diseases among individuals with MetS. These factors contribute to the progression of atherosclerosis^[3]^ and elevate the risks of coronary artery disease, stroke, renal artery stenosis, and peripheral artery disease. Understanding the link between white blood cell (WBC) parameters and metabolic syndrome can aid in identifying high-risk individuals and guiding preventive measures. Various clinical trials and research conducted indicated a relation between hematological parameters (e.g., platelets and WBC indices) and MetS.^[4,5]^ Additionally, insulin resistance and a pro-inflammatory, pro-thrombotic state in MetS lead to metabolic imbalances, including elevated WBCs and platelet count.^[6]^ Managing MetS after its onset requires strict dietary and lifestyle changes. Therefore, utilizing WBC parameters as a diagnostic tool is essential, as it can provide insights into the inflammatory processes involved in the development of insulin resistance and T2DM. This aids in identifying risks and implementing preventive interventions.

Given the alarming consequences of MetS and the anticipated increase in its prevalence among future generations due to unhealthy lifestyle habits, it is imperative to address this syndrome effectively. Our research aims to establish associations between blood markers and MetS, providing valuable insights for researchers to conduct diagnostic tests that can assess an individual’s risk of developing MetS. Additionally, our study aims to recommend effective preventive measures and treatment approaches to delay or mitigate the progression of this syndrome. To the best of our knowledge, several studies have investigated the association between white blood cell parameters and metabolic syndrome, however, no systematic review and meta-analysis has been conducted so far.

## 2. Methods

### 2.1. Literature search and strategy

A systematic review and meta-analysis were conducted according to the Preferred Reporting Items for Systematic Reviews and Meta-Analyses (PRISMA) guidelines.^[7]^ The literature from PubMed, EMBASE, Scopus and Cochrane Library databases from January 2006 to January 2023 was systematically searched using an extensive search strategy in the Supplementary file (Table S1, Supplemental Digital Content, http://links.lww.com/MD/L812). The search strategy was created by merging the following Medical Subject Headings descriptors: “metabolic syndrome,” “leukocyte,” “leucocyte,” “white blood,” “WBC,” “hematological,” and “complete blood count.” Furthermore, manually searching the reference lists of included studies enhanced the tracking of accessible literature.

### 2.2. Study selection

Articles were independently reviewed for inclusion, and any discrepancies between reviewers were discussed and resolved with another reviewer. Articles that met the following criteria were included: observational studies or Randomized Controlled Trials (RCTs) that examined the relationship between MetS and leukocyte parameters such as total leukocyte count, neutrophil, lymphocyte, basophil, eosinophil, monocyte count, Neutrophil-to-lymphocyte ratio (NLR), monocyte-to-lymphocyte ratio (MLR) and lymphocyte-to-Monocyte ratio (LMR), and studies that included subjects older than 18 years. Finally, abstracts and unpublished studies comparing subjects with and without MetS were excluded.

### 2.3. Data extraction and quality assessment

Two reviewers independently screened all papers acquired through the search based on the predetermined eligibility criteria. The following data were extracted from each study: study population (including age and sex), study design, sample size, study, and the mean and standard deviation of leukocyte parameters in those subjects with and without MetS. The risk of bias was assessed using the Newcastle-Ottawa quality assessment scale for observational studies.^[8]^ Cochrane Risk of Bias tool 2.0 was used to assess RCTs.^[9]^

### 2.4. Statistical analysis

For the meta-analysis, the Cochrane Review Manager software (RevMan 5.4.1) was used to perform the statistical calculations and create the forest plots and funnel plots. Due to the difference in effect size of the included studies, a meta-analysis was performed using the Mantel-Haenszel statistical method via Inverse–Variance random-effects model. The difference between arithmetic means with a 95% confidence interval was used to measure effect size and a *P* value of < .05 was considered significant. Publication bias was checked using funnel plots and asymmetry tests (Begg’s test and Egger’s test).^[10]^ When the *P* value of either Egger’s test or Begg’s test was less than 0.05, publication bias was considered significant. Heterogeneity was analyzed using the Chi-square test and the inconsistency index (*I*^2^). According to the Cochrane Collaboration tool, heterogeneity was classified as unimportant (0–40%), moderate (30–60%), substantial (50–90%) and considerable (75–100%). A *P* value < .05 was considered significant. To ensure the robustness of our findings, we conducted a leave-one-out sensitivity analysis when high heterogeneity was observed. This analysis involved iteratively removing one study at a time.

## 3. Results

### 3.1. Characteristics of the studies

Initially, a total of 6068 articles were identified. Following the removal of duplicates and the evaluation of titles and abstracts, 4302 articles were screened for the eligibility criteria. Ultimately, a systematic review and meta-analysis were conducted on a selected pool of 63 articles. In total, the 63 selected papers (4 RCTs and the rest were observational studies) compared different leukocyte parameter concentrations between 53,368 subjects with MetS and 114,777 controls.

Given the large number of articles found in the search, it was divided into 3 subgroups: articles providing leukocyte parameters data globally without distinction of sex (n = 50); articles with disaggregated data for males (n = 14); and female (n = 14). The detailed characteristics of the selected studies are shown in Table S2, Supplemental Digital Content, http://links.lww.com/MD/L813. Thirty-seven studies^[5,11–46]^ defined MetS according to the third report of the National Cholesterol Education Program-Adult Treatment Panel.^[47]^ Fifteen studies^[6,48–61]^ assessed metabolic syndrome using the International Diabetes Federation criteria.^[62]^ Seven studies^[63–69]^ used the harmonized criteria.^[70]^ Two studies^[71,72]^ used the criteria defined American Heart Association/National Heart, Lung, and Blood Institute Scientific Statement.^[1]^ Hai Yan Lin^[73]^ used Chinese Medical Association criteria set^[74]^ and, finally, Chang-Hsun Hsieh^[75]^ used the criteria proposed from the NHANES III data set.^[76]^

### 3.2. Quantitative analysis of WBC parameters

#### 3.2.1. Total leukocyte count (TLC).

**Figure 1.**
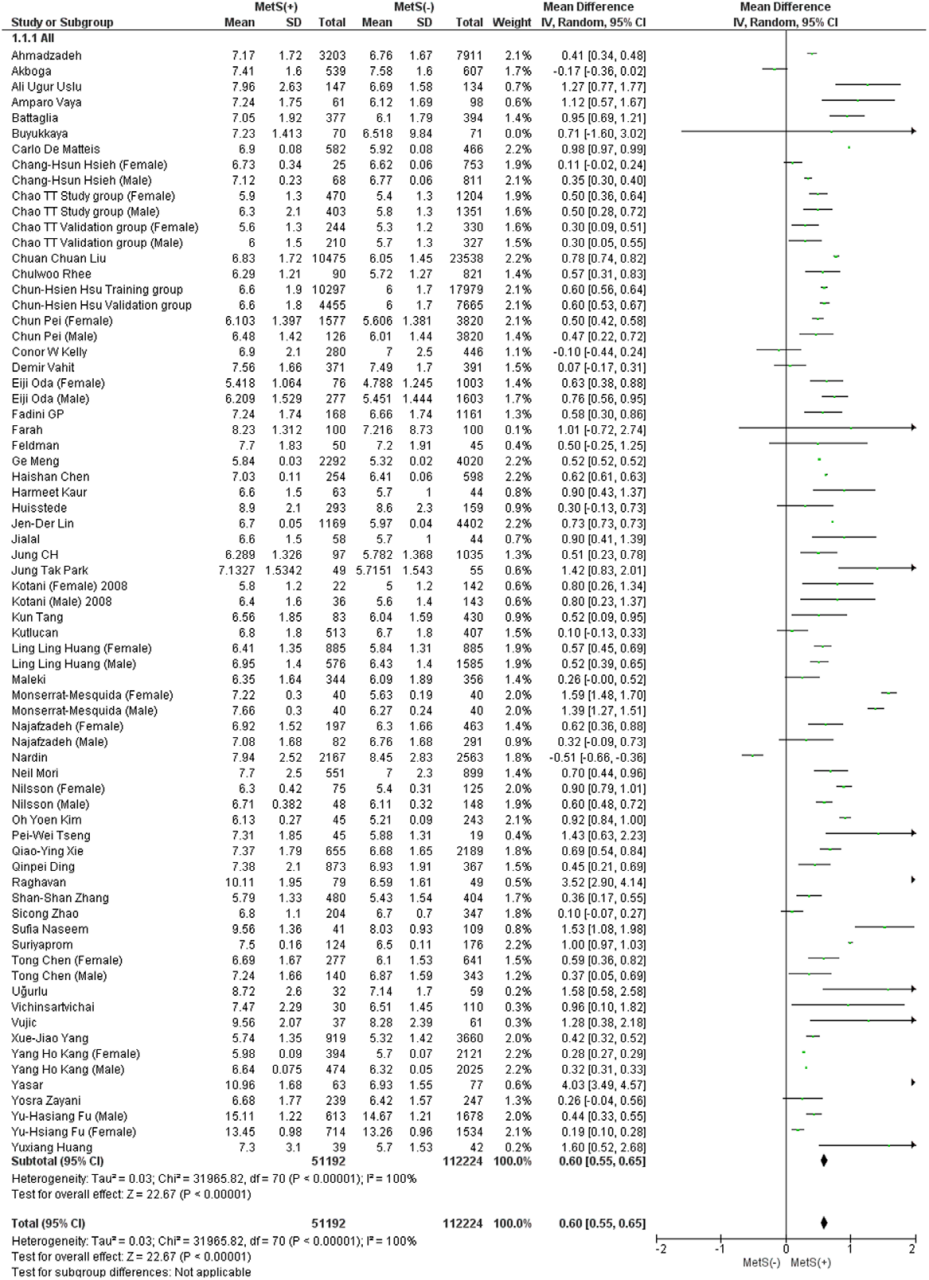
Forest plot analyzing total leukocyte count (×103/mL) in the total population with and without MetS. CI = confidence interval, IV = inverse variance, MetS = metabolic syndrome.

There are 56 papers included in this analysis, of which 12 include female and male subgroups (Fig. 1). Subjects with MetS had a significantly higher mean TLC than those without this syndrome (MD: 0.60 × 10^3^/mL; 95% CI 0.55–0.65; *P* < .00001; *I*^2^ = 100%). Despite conducting a subgroup and leave-one-out sensitivity analysis, the heterogeneity remained high. Upon subgroup analysis, there were no significant subgroup differences between males and females. (MD: 0.55 × 103/mL; 95% CI: 0.49–0.60; *P* = .80) (Figure S1, Supplemental Digital Content, http://links.lww.com/MD/L818). Publication bias was statistically significant for overall TLC (*P* value for Egger: 0.00278; *P* value for Begg: 0.00000) (Table S3, Supplemental Digital Content, http://links.lww.com/MD/L814).

#### 3.2.2. Neutrophil count.

**Figure 2.**
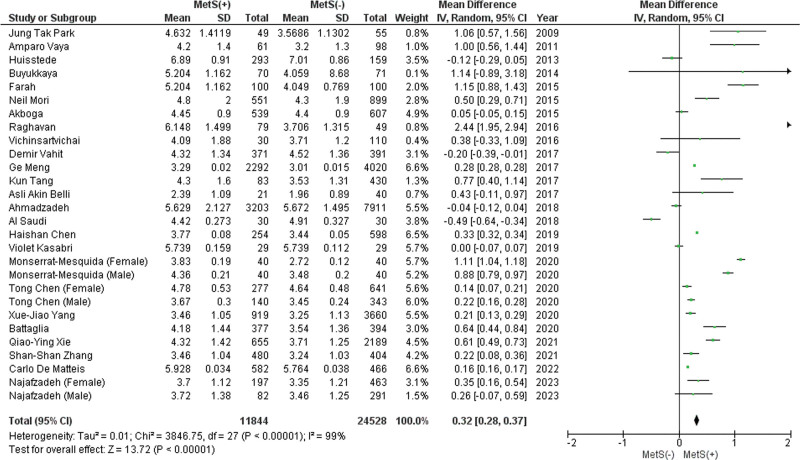
Forest plot analyzing neutrophil count (×103/mL) in the total population with and without MetS. CI = confidence interval, IV = inverse variance, MetS = metabolic syndrome.

A pooled analysis of 25 studies, including 3 studies with male and female subgroups, shows that patients with MetS had significantly higher neutrophil counts (MD: 0.32 × 10^3^/mL; 95% CI 0.28–0.37; *P* < .00001; *I*^2^ = 99%) (Fig. 2). The leave-one-out sensitivity analysis revealed that no study significantly affected the meta-analysis’ heterogeneity. Publication bias was statistically significant (*P* value for Egger: 0.04448; *P* value for Begg: 0.01353) (Table S3, Supplemental Digital Content, http://links.lww.com/MD/L814).

#### 3.2.3. Lymphocyte count.

**Figure 3.**
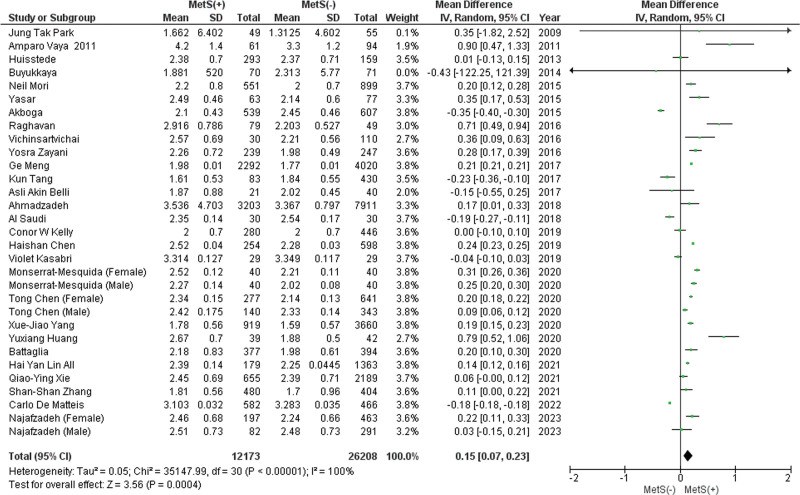
Forest plot analyzing lymphocyte count (×103/mL) in the total population with and without MetS. CI = confidence interval, IV = inverse variance, MetS = metabolic syndrome.

This analysis included 28 studies, of which 3 studies reported male and female data separately and demonstrated that patients with MetS had a significantly higher mean lymphocyte count (MD: 0.15 × 10^3^/mL; 95% CI 0.07–0.23; *P* = .0004; *I*^2^ = 100%) (Fig. 3). A leave-one-out sensitivity analysis revealed that no study had a significant impact on the heterogeneity of the meta-analysis. Publication bias was statistically insignificant (*P* value for Egger: 0.17533; *P* value for Begg: 0.06154) (Table S3, Supplemental Digital Content, http://links.lww.com/MD/L814).

#### 3.2.4. Basophil count.

**Figure 4.**
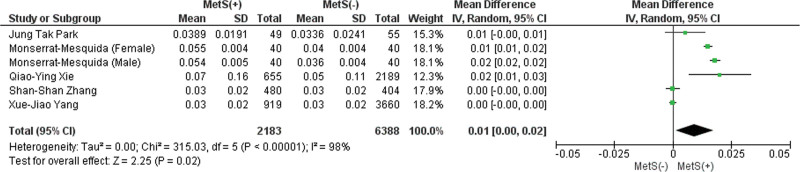
Forest plot analyzing basophil count (×103/mL) in the total population with and without MetS. CI = confidence interval, IV = inverse variance, MetS = metabolic syndrome.

This analysis included 4 studies showing that patients with MetS showed a significantly higher mean basophil count (MD: 0.01 × 10^3^/mL; 95% CI 0.00–0.02; *P* = .02; *I*^2^ = 98%). As a result of the leave-one-out sensitivity analysis, no studies affected the heterogeneity of the meta-analysis substantially (Fig. 4). Publication bias was statistically significant (*P* value for Egger: 0.03901; *P* value for Begg: 0.06029) (Table S3, Supplemental Digital Content, http://links.lww.com/MD/L814).

#### 3.2.5. Eosinophil count.

This analysis included 6 studies, of which 1 study reported male and female subgroups. Patients with MetS showed a non-significantly higher mean eosinophil count (MD: 0.02 × 10^3^/mL; 95% CI −0.01 to 0.05; *P* = .19; *I*^2^ = 96%) (Figure S2, Supplemental Digital Content, http://links.lww.com/MD/L819). Based on the leave-one-out sensitivity analysis, the Monserrat-Mesquida (Female-subgroup)^[65]^ study significantly affected the heterogeneity of the meta-analysis, which was reduced to 63%. However, the results were not significant (*P* = .20) (Figure S3, Supplemental Digital Content, http://links.lww.com/MD/L820). Publication bias was statistically insignificant (*P* value for Egger: 0.06944; *P* value for Begg: 0.07151) (Table S3, Supplemental Digital Content, http://links.lww.com/MD/L814).

#### 3.2.6. Monocyte count.

20 studies (2 studies reported male and female subgroups separately) show that patients with MetS showed a significantly higher mean monocyte count (MD: 0.05 × 10^3^/mL; 95% CI 0.02–0.09; *P* = .003; *I*^2^ = 99%) (Figure S4, Supplemental Digital Content, http://links.lww.com/MD/L821). Upon leave–one out sensitivity analysis, no significant change in heterogeneity was observed. Publication bias was statistically insignificant (*P* value for Egger: 0.14690; *P* value for Begg: 0.05518) (Table S3, Supplemental Digital Content, http://links.lww.com/MD/L814).

#### 3.2.7. NLR.

This analysis includes 11 studies and represents the results obtained when analyzing the NLR in patients with and without MetS. In this case, MetS patients showed a significantly higher mean NLR (MD: 0.24 × 10^3^/mL; 95% CI 0.15–0.33; *P* < .00001; *I*^2^ = 98%) (Figure S5, Supplemental Digital Content, http://links.lww.com/MD/L823). Based on a leave-one-out sensitivity analysis, no study had a substantial influence on the heterogeneity of the meta-analysis, so none was excluded. Publication bias was statistically insignificant (*P* value for Egger: 0.78092; *P* value for Begg: 0.63122) (Table S3, Supplemental Digital Content, http://links.lww.com/MD/L814).

#### 3.2.8. MLR and LMR.

MLR and LMR were pooled analyses in patients with and without MetS. There were significant subgroup differences between the subgroups (MD: −0.04 × 10^3^/mL; 95 % CI: −0.14 to 0.06; *P* = .0003; *I*^2^ = 92.4%) (Figure S6, Supplemental Digital Content, http://links.lww.com/MD/L824). In the case of MLR, patients with MetS had a mean ratio that was non-significantly higher (MD: 0.06; 95% CI −0.05 to 0.17; *P* = .27; *I*^2^ = 100%). In contrast, patients with MetS had a significantly lower mean LMR ratio (MD: −0.52; 95% CI −0.81 to −0.23; *P* = .0005; *I*^2^ = 52%) (Figure S6, Supplemental Digital Content, http://links.lww.com/MD/L824). The sensitivity analysis revealed that no study had a substantial effect on the heterogeneity of the meta-analysis, so none were omitted. Publication bias was statistically insignificant for MLR (*P* value for Egger: 0.26665; *P* value for Begg: 1.00000) (Table S3, Supplemental Digital Content, http://links.lww.com/MD/L814). Publication bias was ineligible for LMR since only 2 studies were involved.

### 3.3. Quality assessment

In the quality assessment using the Newcastle-Ottawa scale, most studies were of high quality. However, 26 of the studies displayed a high risk of publication bias, and only 1 study demonstrated a very high risk of bias, as shown in Tables S4–S6, Supplemental Digital Content, http://links.lww.com/MD/L815; http://links.lww.com/MD/L816; http://links.lww.com/MD/L817. The Cochrane tool for assessing the risk of bias in RCTs indicated a low risk of bias across all studies (Figure S7, Supplemental Digital Content, http://links.lww.com/MD/L825).

## 4. Discussion

To our knowledge, this is the first and largest meta-analysis including over 160,000 participants from 63 studies that has aimed to review the relationship between the total and differential white blood cell counts in patients with MetS. Our results revealed that the TLC, neutrophil, lymphocyte, monocyte, basophil counts and NLR, were significantly higher in participants with metabolic syndrome when compared with the control group. There were no significant differences in the eosinophil count and MLR between patients with and without MetS, however, the LMR of patients with MetS was lower.

Our results revealed that patients with Metabolic syndrome had a significantly higher mean TLC than those without the syndrome. This could be explained by the fact that visceral adiposity plays a key role in the pathogenesis of the syndrome. Studies have shown that visceral adiposity is one of the clinical indicators of MetS creating a subclinical chronic inflammatory state.^[77]^ Visceral adipose tissue produces cytokines including interleukin-1 and tumor necrosis factor-α (TN-α) which contributes to this chronic inflammatory state by stimulating the production of white blood cells.^[78]^ Hence, high leukocyte counts can be an indicator of some diseases like metabolic syndrome.^[79]^ Studies have also shown that visceral adipose tissue acts as an endocrine organ disrupting normal hormonal homeostasis and thus contributing to insulin resistance. It is also known that insulin, a major regulatory hormone in Metabolic syndrome plays a role in increased circulation of WBCs.^[80,81]^ In addition, increased resistance to insulin gives rise to higher serum viscosity, produces a prothrombotic state, and further increases pro-inflammatory cytokine release from the adipose tissue, all of which contributes to increased risk of cardiovascular diseases and T2DM.

Our analysis also revealed that the mean neutrophil count in patients with MetS was significantly higher than those without. This finding can be explained by Kaur et al^[11]^ who elucidated that the total increase in TLC could be attributed primarily to an increase in the number of neutrophils.^[79]^ They utilized the homeostasis model assessment of insulin resistance; which employed fasting recordings of blood glucose and insulin concentrations; as a surrogate marker for quantifying insulin resistance as well as hs-CRP (highly sensitive C reactive protein) as a marker of inflammation. Their analysis revealed that the increased neutrophil count significantly correlated with elevated levels of both biomarkers.^[11]^ Thus, neutrophil counts can prove to be more useful markers for metabolic syndrome.^]^

NLR can be calculated by dividing the total number of neutrophils by the total number of lymphocytes. This has recently been used as an important inflammatory biomarker in multiple diseases specifically linked to MetS which include atherosclerosis, myocardial infarction, and diabetes mellitus.^[82,83]^ Elevated ratios have also been incriminated in poor prognostic outcomes of said diseases. This is consistent with the results of our analysis which revealed that NLR of patients with MetS was significantly higher than the control group of participants. A study by Liu et al^[44]^ not only revealed that patients with MetS had a significantly higher NLR than patients without but that patients with MetS who fulfilled all 5 criteria had a higher NLR score than patients who fulfilled only 3 out of 5 of the clinical criteria. Thus, NLR is not only an important biomarker for the presence of the syndrome but also for predicting the severity of it.

Multiple studies^[84,85]^ have implicated raised eosinophil count in the pathogenesis of atherogenic dyslipidemias which is among the diagnostic criteria of MetS. Lin et al^[86]^ conducted a massive study encompassing over 10,000 participants over a period of 10 years, the result of which showed that high eosinophil count was associated with raised triglyceride levels and reduced HDL-C levels. Moreover, it also revealed that the odds ratio of MetS increased significantly with ascending eosinophil count. However, our results revealed a non-significant association between the eosinophil count and the presence of MetS. Thus, further studies are needed to draw plausible conclusions regarding this association.

Our findings revealed no significant differences in TLC count between males and females with Metabolic Syndrome (MetS). This aligns with existing literature, where a study conducted in Africa^[87]^ also observed increased WBC counts with a rise in the number of MetS components for both genders, though statistical significance was not achieved. Similarly, Tian et al^[88]^ reported an association, but the difference in WBC counts was minimal, possibly due to their smaller sample size. Additionally, Jiang et al^[89]^ demonstrated the presence of this association even after adjusting for known risk factors of glucose status change, such as age, obesity, history of hypertension, family history of diabetes, history of dyslipidemia, and history of coronary heart disease. These results suggest that an elevated WBC count serves as an independent risk factor for MetS. However, the exact mechanism underlying this increase in WBC counts in MetS patients remains unclear, although insulin resistance might play a partial role.^[90]^

Our study is not without its limitations. A major concern is heterogeneity among studies. Even after performing a sensitivity analysis and subgroup analysis, it did not significantly reduce. This could have resulted from the inclusion of a wide range of age groups with some studies taking school-aged children^[91]^ while others include adults or elderly.^[14,63]^ Karakaş Uğurlu et al^[26]^ looked for metabolic syndrome findings in schizophrenic patients only,^[90]^ and Rhee et al^[39]^ investigated this relation in military aviators.^[91]^ Due to inherent differences in metabolism, these populations are expected to exhibit variations in results compared to the general population. Comorbidities and smoking addiction were not considered, introducing potential confounding bias^[86,87]^ Moreover, prior inflammatory diseases should have been ruled out. Furthermore, our study predominantly focused on research conducted with the Chinese population. Therefore, it is imperative to conduct further research in other countries and include it in meta-analyses to obtain a more comprehensive understanding of this topic across diverse populations.

## 5. Conclusion

The results of our analysis revealed that patients with metabolic syndrome had a significantly elevated mean TLC, neutrophil count, basophil count, lymphocyte count and a high NLR in comparison to patients without this syndrome, suggesting a subclinical inflammatory state to be the harbinger of this syndrome. These lab parameters which are included in the routine full blood count can help clinicians in the early diagnosis of the syndrome when coupled with the existing diagnostic criteria. This can help curb the tide of the rapidly increasing burden of metabolic disorders which subsequently lead to far more severe diseases including CVAs, T2DM and myocardial infarctions.

## Author contributions

**Conceptualization:** Aysal Mahmood, Abraish Ali, Muhammad Zain Farooq, Hadi Farhat.

**Data curation:** Aysal Mahmood, Hoorain Haider, Saba Samad, Danisha Kumar, Aimen Perwaiz, Rabeea Mushtaq.

**Formal analysis:** Aysal Mahmood, Danisha Kumar, Abraish Ali.

**Investigation:** Aysal Mahmood, Saba Samad, Danisha Kumar, Aimen Perwaiz, Rabeea Mushtaq.

**Methodology:** Aysal Mahmood, Hoorain Haider, Saba Samad, Danisha Kumar, Aimen Perwaiz, Rabeea Mushtaq.

**Project administration:** Hoorain Haider, Abraish Ali, Hadi Farhat.

**Resources:** Hoorain Haider, Danisha Kumar, Aimen Perwaiz, Rabeea Mushtaq, Hadi Farhat.

**Software:** Aysal Mahmood, Aimen Perwaiz, Hadi Farhat.

**Supervision:** Aysal Mahmood, Abraish Ali, Muhammad Zain Farooq.

**Validation:** Rabeea Mushtaq, Abraish Ali, Muhammad Zain Farooq.

**Visualization:** Aimen Perwaiz, Rabeea Mushtaq, Abraish Ali, Muhammad Zain Farooq, Hadi Farhat.

**Writing – original draft:** Aysal Mahmood, Hoorain Haider, Saba Samad, Aimen Perwaiz, Rabeea Mushtaq.

**Writing – review & editing:** Hoorain Haider, Saba Samad, Abraish Ali, Muhammad Zain Farooq, Hadi Farhat.

## Supplementary Material













**Figure:**
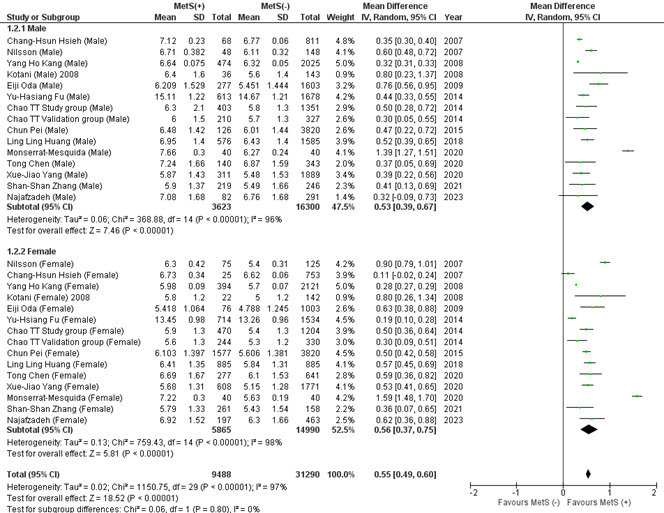


**Figure:**
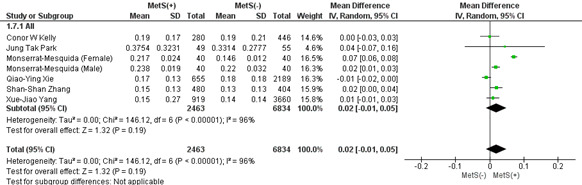


**Figure:**
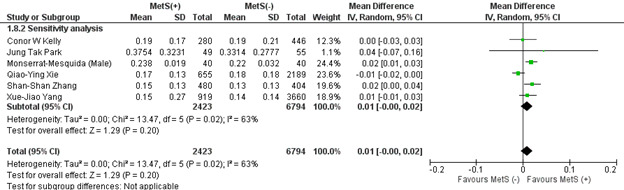


**Figure:**
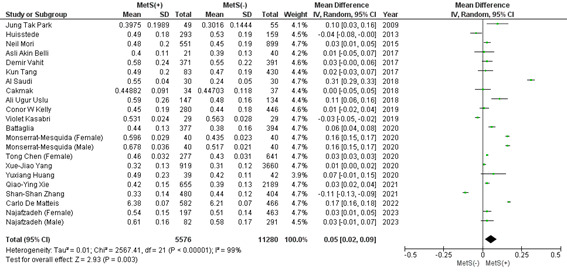


**Figure:**
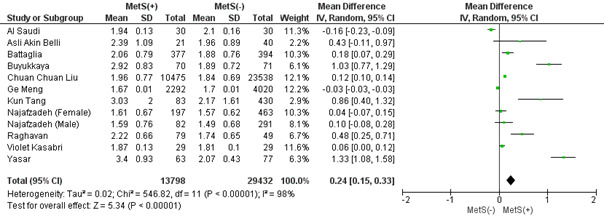


**Figure:**
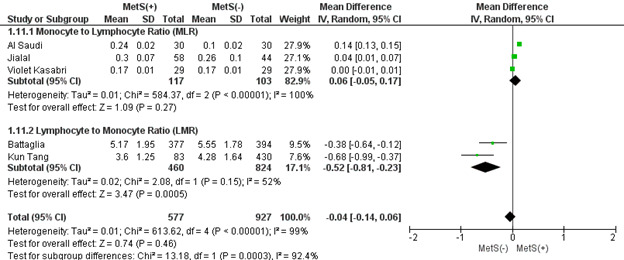


**Figure:**