# Supplementary material for: Association of white blood cell parameters with metabolic syndrome: A systematic review and meta-analysis of 168,000 patients
Source: Medicine (Baltimore). 2024 Mar 8;103(10):e37331. doi: 10.1097/MD.0000000000037331 (PMC10919507; doi:10.1097/MD.0000000000037331)
Supplement: Supplementary file 1 [file medi-103-e37331-s001.docx]

**Supplemental Table 1.** Intraocular lens types implanted in the study cohort

| **Lens name** | **Frequency** | **Percentage** |
| --- | --- | --- |
| **All multifocal lenses** | **38,828** | **77.0%** |
| Tecnis Multifocal +2.75 Add ZKB00^1^ | 18,963 | 37.6% |
| Tecnis Symfony ZXR00^1^ | 7,926 | 15.7% |
| Tecnis Symfony Toric ZXT^1^ | 2,244 | 4.5% |
| Lenstec SBL-2^2^ | 1,955 | 3.9% |
| Lentis Mplus^X^ MF30^3^ | 1,507 | 3.0% |
| Lentis Mplus MF30^3^ | 1,379 | 2.7% |
| Tecnis Multifocal +3.25 Add ZLB00^1^ | 1,319 | 2.6% |
| AT LARA 829MP^4^ | 1,282 | 2.5% |
| Other multifocal lenses implanted in less than 1000 eyes per model | 2,253 | 4.5% |
| **All monofocal lenses** | **11,571** | **23.0%** |
| Tecnis Monofocal ZCB00^1^ | 6,846 | 13.6% |
| Tecnis Monofocal PCB00^1^ | 1,862 | 3.7% |
| Tecnis Monofocal Toric ZCT^1^ | 1,803 | 3.6% |
| Other monofocal lenses implanted in less than 1000 eyes per model | 1,060 | 2.1% |

^1^Johnson & Johnson Vision Care, Inc, Santa Ana, CA

^2^ Lenstec, Inc., Christ Church, Barbados

^3^ Teleon Surgical B.V., Spankeren, Netherlands; formerly manufactured by Oculentis GmbH, Berlin, Germany

^4^ Carl Zeiss Meditec AG, Jena, Germany
