# Supplementary material for: Association of white blood cell parameters with metabolic syndrome: A systematic review and meta-analysis of 168,000 patients
Source: Medicine (Baltimore). 2024 Mar 8;103(10):e37331. doi: 10.1097/MD.0000000000037331 (PMC10919507; doi:10.1097/MD.0000000000037331)
Supplement: Supplementary file 2 [file medi-103-e37331-s002.docx]

| **Author, year country** | **Study Type** | **Number of participants** | | **MetS criteria** | **Mean age (years)** | |
| --- | --- | --- | --- | --- | --- | --- |
|  |  |  |  |  |  |  |
|  |  | **MetS** | **Without MetS** |  | **MetS** | **Without MetS** |
| Harmeet Kaur, 2013, USA | Cross-sectional study | 63 | 44 | ATP III | 53 (11) | 50 (12) |
| Yang Ho Kang, 2008, Korea | Prospective cross-sectional | M=474 | M= 2025 | ATP III | M= 48.3 | |
|  |  | W=394 | W= 2121 |  | W= 47.8 | |
| G Nilsson, 2007, Sweden | Prospective cross-sectional | M= 48 | M= 148 | ATP III | >75 | |
|  |  | W=75 | W=125 |  |  |  |
| Xue-Jiao Yang, 2020, China | Prospective cross-sectional | M= 311 | M=1889 | ATP III | >60 years | |
|  |  | W=608 | W=1771 |  |  |  |
| Hai Yan Lin, 2021, China | Prospective cohort | 179 | 1363 | Chinese Medical Association | 45.0 (Median) IQR (44.3-46.7) | 44.9 (Median) IQR (44.2-45.6) |
| Chao TT, 2014, Taiwan | Prospective longitudinal study | Study-M= 403 | Study-M= 1351 | Harmonized criteria | 67.3 | 68.8 |
|  |  | Study-F= 470 | Study-F= 1204 |  | 65.9 | 67.2 |
|  |  | Validation-M= 210 | Validation-M= 327 |  | 65.5 | 66.9 |
|  |  | Validation-F= 244 | Validation-F= 330 |  | 64.8 | 65.8 |
| Jung CH, 2013, Korea | Cross-sectional | 97 | 1035 | ATP III | 50.6 | 49.3 |
| Suriyaprom, 2019, Thailand | Case-control study | 124 | 176 | ATP III | Median 42 (39-43) | Median 40 (38-42) |
| Jialal, 2019, USA | Cross-sectional | 58 | 44 | ATP III | 52.9 ± 10.5 | 49.9 ± 11.7 |
| Mehmet Kadri Akboga, 2015, Turkey | Prospective cohort | 539 | 607 | ATP III | 58.9 ± 10.8 | 55.4 ± 11.8 |
| Dehghani, 2016, Iran | Prospective cohort | 206 | 125 | ATP III | 60.4±11.8 | 59.5±13.6 |
| Huisstede, 2013, Netherlands | Cross-sectional | 293 | 159 | ATP III | 43 | 37 |
| Haishan Chen, 2019, China | Prospective cross-sectional | 254 | 598 | ATP III | 56.48±0.53 | 47.67±0.45 |
| Amparo Vaya, 2011, Spain | Case control study | 61 | 98 | ATP III | 51.72 ± 11.97 | 49.99 ± 9.54 |
| Ali Maleki, 2014, Iran | Cross-sectional | 344 | 356 | ATP III | 55.51 ± 10.88 | 54.37 ± 13.09 |
| Cakmak, 2018, Turkey | Cross-sectional | 34 | 37 | ATP III | 26.06 ± 5.43 | 23.51 ± 5.25 |
| Kutlucan, 2012, Turkey | Prospective cohort | 513 | 407 | ATP III | 54 ± 11 | 50 ± 13 |
| Uğurlu, 2016, Turkey | Prospective cross-sectional | 32 | 59 | ATP III | 43.0 ± 10.6 | 31.7 ± 8.6 |
| Sufia Naseem, 2019, India | Prospective cohort | 41 | 109 | IDF | 60.37±6.86 | 57.92±7.20 |
| Ahmadzadeh, 2018, Iran | Clinical trial | 3203 | 7911 | IDF | 41.42±9.96 | 36.47±9.64 |
| Qiao-Ying Xie, 2021, China | Prospective cohort | 655 | 2,189 | IDF | 26.1 | 25.7 |
| Najafzadeh, 2023, Iran | Cross-sectional | M= 82 | M= 291 | ATP III | 51.90 ± 13.84 | 45.48 ± 16.21 |
|  |  | F= 197 | F= 463 |  | 53.33 ± 11.72 | 40.29 ± 14.07 |
| Sicong Zhao, 2016, China | Prospective cross-sectional | 204 | 347 | ATP III | 63.1±10.6 | 67.3±9.9 |
| Ali Ugur Uslu, 2018, Turkey | Retrospective cross-sectional | 147 | 134 | ATP III | 47 | 44 |
| Feldman, 2014, France | Prospective cohort | 50 | 45 | IDF | Median 61.5 (IQR=54.8-69) | 59.3 (53.3-66.2) |
| Neil Mori, 2015, USA | Clinical trial | 551 | 899 | AHA/National Heart, Lung, and Blood Institute Scientific Statement | 41.7 ± 10.5 | 39.9 ± 11.5 |
| Pei-Wei Tseng, 2017, Taiwan | Prospective cross-sectional | 45 | 19 | IDF | 71.00 (67.00-76.00) | 68.00 (66.00-80.00) |
| Vichinsartvichai, 2016, Thailand | Prospective cross-sectional | 30 | 110 | Harmonized criteria | 52.0 ±8.0 | 49.5 ±7.2 |
| Kun Tang, 2017, China | Retrospective cohort | 83 | 430 | ATP III | 53.1 ± 10.2 | 52.1 ± 11.1 |
| Yuxiang Huang, 2020, China | Cross sectional | 39 | 42 | ATP III | 43 ± 12 | 31.5 ± 10.75 |
| Stefano Battaglia, 2020, Italy | Prospective cohort | 377 | 394 | IDF | 61.80±12.08 | 51.12±14.90 |
| Tong Chen, 2020, China | Cross sectional | M= 140 | M= 343 | IDF | 57.2 ± 10.5 | 54.8 ± 13.5 |
|  |  | F= 277 | F= 641 |  | 60.7 ± 10.0 | 52.6 ± 12.7 |
| Monserrat-Mesquida, 2020, Spain | Prospective observational | M= 40 | M= 40 | Harmonized criteria | 63.8 ± 0.8 | 65.7 ± 0.8 |
|  |  | F= 40 | F= 40 |  | 64.1 ± 0.5 | 66.8 ± 0.8 |
| Raghavan, 2016, India | Case control | 79 | 49 | IDF | 52.29 ± 10.30 | 42.41 ± 13.22 |
| Yosra Zayani, 2016, Tunisia | Case-control | 239 | 247 | ATP III | 51.73 ± 7.17 | 51.17 ± 12.33 |
| Yu-Hsiang Fu, 2014, China | Prospective longitudinal | M= 613 | M= 1678 | Harmonized criteria | 66.7 ± 5.6 | 68.4 ± 5.1 |
|  |  | F= 714 | F= 1534 |  | 65.5 ± 4.4 | 66.9 ± 4.0 |
| Fadini GP, 2012, Italy | Prospective cohort | 168 | 1161 | ATP III | 45.4 ± 6.9 | 40.7 ± 7.2 |
| Oh Yoen Kim, 2013, Korea | Longitudinal observational | 45 | 243 | ATP III | 30-69 |  |
| Buyukkaya, 2014, Turkey | Prospective observational | 70 | 71 | ATP III | 48 + 10 | 47 + 13 |
| Conor W Kelly, 2019, USA | Clinical trial | 280 | 446 | American Heart Association/National Heart, Lung, and  Blood Institute Scientific Statement | 39.0 ± 10.7 | 37.6 ± 11.6 |
| Ling Ling Huang, 2018, China | Prospective cross-sectional | M= 576 | M=1585 | ATP III | 51.39±12.21 | 54.61±13.79 |
|  |  | F= 885 | F= 885 |  | 59.78±12.34 | 55.70±12.97 |
| Carlo De Matteis, 2022, Italy | Prospective cross-sectional | 582 | 466 | IDF | 60.71 ± 0.48 | 50.01 ± 0.73 |
| Demir Vahit, 2017, Turkey | Retrospective observational | 371 | 391 | ATP III | 57.4 ± 8.8 | 56.3 ± 9.1 |
| Kazuhiko Kotani, 2008, Japan | Prospective observational | M= 36 | M= 143 | ATP III | M= 51 ± 9 | |
|  |  | F= 22 | F= 142 |  | W= 49 ± 9 | |
| Chulwoo Rhee, 2015, Korea | Prospective cross sectional | 90 | 821 | ATP III |  |  |
| Chang-Hsun Hsieh, 2007, Taiwan | Prospective observational | M= 68 | M= 811 | NHANES IV data set | M= 16.44 ± 1.8 | M= 16.5 ± 1.67 |
|  |  | F= 25 | F= 753 |  | W= 16.48 ± 1.71 | W= 16.83 ± 1.70 |
| Chun Pei, 2015, Taiwan | Cross sectional study | M= 1246 | M= 3820 | Harmonized criteria | M= 65.1 ± 5.4, | M =64.8 ± 5.2, |
|  |  | F= 1577 | F= 3820 |  | F= 23.068 ± 2.842 | F= 63.4 ± 4.1 |
| Qinpei Ding, 2021, China | Retrospective case control study | 873 | 367 | IDF | 53.36 ± 13.22 | 53.83 ± 12.46 |
| Asli Akin Belli, 2017, Turkey | Case-control study | 21 | 40 | IDF | 33-78 |  |
| Nardin, 2019, Italy | Prospective observational | 2167 | 2563 | ATP III | 68.48 ± 10.42 | 67.07 ± 11.94 |
| Eiji Oda, 2009, Japan | Prospective cross-sectional | M= 277 | M= 1603 | ATP III | 52.9 ± 8.5 | 50.9 ± 9.2 |
|  |  | F = 76 | F= 1003 |  | 57.1 ± 9.7 | 50.4 ± 9.4 |
| Jung Tak Park, 2009, Korea | Prospective cross-sectional | 49 | 55 | ATP III | 53.0 ± 10.2 | 50.4 ± 10.4 |
| Shan-Shan Zhang, 2021, China | Prospective cross-sectional | All= 480 | All= 404 | ATP III | 66.20 ± 5.14 | 66.62 ± 5.46 |
|  |  | M= 219 | M= 246 |  |  |  |
|  |  | F= 261 | F= 158 |  |  |  |
| Amparo Vaya, 2011, Spain | Case-control study | 61 | 94 | ATP III | 51 ± 11 | 50 ± 10 |
| T Vujic, 2016, Serbia | Prospective cross-sectional | 37 | 61 | IDF | 63.43 ± 8.46 | 65.70 ± 8.84 |
| Z Yasar, 2015, Turkey | Retrospective cohort | 63 | 77 | IDF | 64.79 ± 9.09 | 65.47 ± 10.81 |
| Ge Meng, 2017, China | Prospective cohort | 2292 | 4020 | Harmonized criteria | Median 52.7 IQR (52.3, 53.1) | Median 48.9 IQR (48.7, 49.2) |
| Chuan Chuan Liu, 2019, Taiwan | Prospective cohort | 10475 | 23538 | ATP III | 50.46 ± 11.09 | 45.56 ± 11.08 |
| Farah, 2015, Israel | Randomized Controlled Trial | 100 | 100 | ATP III | 49±12 | 48±14 |
| Violet Kasabri, 2019, Jordan | Prospective cross-sectional | 29 | 29 | IDF | 49.07 ± 2.00 | 44.39 ± 2.05 |
|  |  |  |  |  |  |  |
| Al Saudi, 2018, Jordan | Prospective cross-sectional | 30 | 30 | IDF | 44.79 | 30.48 |
|  |  |  |  |  |  |  |
| Chun-Hsien Hsu, 2021, China | Cross sectional | Training group = 10,297 | Training group = 17,979 | Harmonized criteria | 66.4 ± 5.8 | 65.3 ± 5.5 |
|  |  | Validation Group = 4,455 | Validation Group = 7,664 |  | 66.4 ± 5.9 | 65.4 ± 5.5 |
| Jen-Der Lin, 2006, China | Retrospective cohort | 1665 | 5238 | ATP III | 52.7 ± 7.6 | 48.5 ± 9.3 |

Table S1: Basic characteristics of the included studies.

(ATP-III: Adult Treatment Panel; AHA: American Heart Association; IDF: International Diabetes Federation; MetS: Metabolic Syndrome; NHANES: National Health and Nutrition Examination Survey)
