# Supplementary material for: Association of white blood cell parameters with metabolic syndrome: A systematic review and meta-analysis of 168,000 patients
Source: Medicine (Baltimore). 2024 Mar 8;103(10):e37331. doi: 10.1097/MD.0000000000037331 (PMC10919507; doi:10.1097/MD.0000000000037331)
Supplement: Supplementary file 3 [file medi-103-e37331-s004.docx]

| **Outcomes** | **Egger's Regression p-value (2-tailed)** | **Begg’s and Mazumdar rank correlation** |
| --- | --- | --- |
| Total Leukocyte Count (TLC). | 0.00278 | 0.00000 |
| Neutrophil count. | 0.04448 | 0.01353 |
| Lymphocyte count. | 0.17533 | 0.06154 |
| Basophil count. | 0.03901 | 0.06029 |
| Eosinophil count. | 0.06944 | 0.07151 |
| Monocyte count. | 0.14690 | 0.05518 |
| Neutrophil-to-lymphocyte ratio (NLR). | 0.78092 | 0.63122 |
| Monocyte-Lymphocyte | 0.12371 | 0.22067 |
| Monocyte-to-lymphocyte ratio (MLR) | 0.26665 | 1.00000 |
| Lymphocyte-to-Monocyte ratio (LMR). | N//A (only 2 studies) | N/A (only 2 studies) |
| WBC female | 0.16753 | 0.00215 |
| WBC male | 0.14658 | 0.00109 |

Table S6: Egger’s regression and Begg’s and Mazumdar rank correlation for all outcomes.
