# Supplementary material for: Association of white blood cell parameters with metabolic syndrome: A systematic review and meta-analysis of 168,000 patients
Source: Medicine (Baltimore). 2024 Mar 8;103(10):e37331. doi: 10.1097/MD.0000000000037331 (PMC10919507; doi:10.1097/MD.0000000000037331)
Supplement: Supplementary file 4 [file medi-103-e37331-s010.docx]

| **Study (Year)** | **Selection** | | | | **Comparability** | | **Outcome** | | | **NOS Score** |
| --- | --- | --- | --- | --- | --- | --- | --- | --- | --- | --- |
|  | **Representativeness of the exposed cohort** | **Selection of the non-exposed cohort** | **Ascertainment of exposure** | **Demonstration that outcome of interest was not present at start of study** | **Comparability of cohorts based on the design or analysis (main factor)** | **Comparability of cohorts based on the design or analysis (additional factor)** | **Assessment of outcome** | **Was follow-up long enough for outcomes to occur** | **Adequacy of follow up of cohorts** |  |
| Harmeet Kaur, 2013 | * | * |  | * | * | * |  |  |  | 5 |
| Yang Ho Kang, 2008 | * | * | * | * | * | * | * |  |  | 7 |
| G Nilsson, 2007 | * | * | * | * | * | * | * |  |  | 7 |
| Xue-Jiao Yang, 2020 | * | * | * | * | * | * | * |  |  | 7 |
| Hai Yan Lin, 2021 | * | * | * | * | * | * | * | * | * | 9 |
| Chao TT, 2014 | * | * | * | * | * |  | * | * | * | 8 |
| Jung CH, 2013 | * | * | * | * | * | * | * |  |  | 7 |
| Jialal, 2019 | * |  |  |  |  |  | * |  |  | 2 |
| Mehmet Kadri Akboga, 2015 | * | * | * | * | * |  | * |  |  | 6 |
| Dehghani, 2016, | * | * | * | * | * | * | * | * | * | 9 |
| Huisstede, 2013 | * | * | * | * | * |  | * |  |  | 6 |
| Haishan Chen, 2019 | * | * | * | * | * | * | * |  |  | 7 |
| Ali Maleki, 2014 | * | * | * | * |  |  | * |  |  | 5 |
| Cakmak, 2018 | * | * | * | * | * | * | * |  |  | 7 |
| Kutlucan, 2012 | * | * | * | * | * |  | * |  |  | 6 |
| Uğurlu, 2016 | * | * | * | * | * |  | * |  |  | 6 |
| Qiao-Ying Xie, 2021 | * | * | * | * |  |  | * |  |  | 5 |
| Najafzadeh, 2023 | * | * | * | * |  |  | * |  |  | 5 |
| Sicong Zhao, 2016 | * | * | * | * | * |  | * |  |  | 6 |
| Ali Ugur Uslu, 2018 | * | * | * | * | * |  | * |  |  | 6 |
| Feldman, 2014 | * | * | * | * |  |  | * | * |  | 6 |
| Pei-Wei Tseng, 2017 | * | * | * | * | * |  | * |  |  | 6 |
| Vichinsartvichai, 2016 | * | * | * | * | * |  | * |  |  | 6 |
| Kun Tang, 2017 | * | * | * | * | * |  | * |  |  | 6 |
| Yuxiang Huang, 2020 | * | * | * | * | * | * | * |  |  | 7 |
| Stefano Battaglia, 2020 | * | * | * | * | * |  | * |  |  | 6 |
| Tong Chen, 2020 | * | * | * | * | * | * | * |  |  | 7 |
| Monserrat-Mesquida, 2020 | * | * | * | * | * | * | * |  |  | 7 |
| Yu-Hsiang Fu, 2014 | * | * | * | * | * |  | * | * | * | 8 |
| Fadini GP, 2012 | * | * | * | * | * | * | * | * |  | 8 |
| Oh Yoen Kim, 2013 | * | * | * | * | * | * | * | * | * | 9 |
| Buyukkaya, 2014 | * | * | * | * | * |  | * |  |  | 6 |
| Ling Ling Huang, 2018 | * | * | * | * | * | * | * |  |  | 7 |
| Carlo De Matteis, 2022, | * | * | * | * | * |  | * |  |  | 6 |
| Demir Vahit, 2017 | * | * | * | * | * | * | * |  |  | 7 |
| Kazuhiko Kotani, 2008 | * |  | * | * | * | * |  |  |  | 5 |
| Chulwoo Rhee, 2015 | * | * | * | * | * | * | * |  |  | 7 |
| Chang-Hsun Hsieh, 2007 | * | * | * | * | * | * | * |  |  | 7 |
| Chun Pei, 2015 | * | * | * | * | * | * | * | * | * | 9 |
| Nardin, 2019 | * | * | * | * | * | * | * |  |  | 7 |
| Eiji Oda, 2009 | * | * |  | * | * | * | * |  |  | 6 |
| Jung Tak Park, 2009 | * | * | * | * | * | * | * |  |  | 7 |
| Shan-Shan Zhang, 2021 | * | * | * | * | * | * | * |  |  | 7 |
| T Vujic, 2016 | * | * | * | * | * |  | * |  |  | 6 |
| Z Yasar, 2015 | * | * | * | * | * |  | * | * |  | 7 |
| Ge Meng, 2017 | * | * | * | * | * | * | * |  |  | 7 |
| Chuan Chuan Liu, 2019 | * | * | * | * | * | * | * |  |  | 7 |
| Violet Kasabri, 2019 | * | * | * | * | * | * | * |  |  | 7 |
| Al Saudi, 2018 | * | * | * | * | * |  | * |  |  | 6 |
| Chun-Hsien Hsu, 2021 | * | * | * | * | * | * | * | * | * | 9 |
| Jen-Der Lin, 2006 | * | * | * | * |  |  | * |  |  | 5 |

Table S3: Newcastle-Ottawa Scale for cohorts and cross-sectional studies.
