# Supplementary material for: Association of white blood cell parameters with metabolic syndrome: A systematic review and meta-analysis of 168,000 patients
Source: Medicine (Baltimore). 2024 Mar 8;103(10):e37331. doi: 10.1097/MD.0000000000037331 (PMC10919507; doi:10.1097/MD.0000000000037331)
Supplement: Supplementary file 5 [file medi-103-e37331-s011.docx]

| **Study (Year)** | **Selection** | | | | **Comparability** | | **Exposure** | | | **NOS Score** |
| --- | --- | --- | --- | --- | --- | --- | --- | --- | --- | --- |
|  | **Is the case definition adequate?** | **Representativeness of the cases** | **Selection of Controls** | **Definition of Controls** | **Comparability of cases and controls on the basis of the design or analysis (main factor)** | **Comparability of cases and controls on the basis of the design or analysis (additional factor)** | **Ascertainment of exposure** | **Same method of ascertainment for cases and controls** | **Non-Response rate** |  |
| Sufia Naseem, 2019 | * | * | * | * |  |  | * | * |  | 6 |
| Suriyaprom, 2019 | * | * | * | * | * | * | * | * | * | 9 |
| Amparo Vaya, 2011 | * | * | * | * |  |  | * | * |  | 6 |
| Amparo Vaya, 2011 | * | * | * | * |  |  | * | * |  | 6 |
| Qinpei Ding, 2021 | * | * | * | * | * | * | * | * |  | 8 |
| Asli Akin Belli, 2017 | * | * | * | * | * | * | * | * |  | 8 |
| Raghavan, 2016 | * | * | * | * |  |  | * | * |  | 6 |
| Yosra Zayani, 2016 | * | * | * | * | * | * | * | * |  | 8 |

Table S4: New-castle Ottawa scale for case-control studies
