# Supplementary material for: Association of white blood cell parameters with metabolic syndrome: A systematic review and meta-analysis of 168,000 patients
Source: Medicine (Baltimore). 2024 Mar 8;103(10):e37331. doi: 10.1097/MD.0000000000037331 (PMC10919507; doi:10.1097/MD.0000000000037331)
Supplement: Supplementary file 6 [file medi-103-e37331-s012.docx]

| **Study (Year)** | **Randomization process** | **Deviations from intended interventions** | **Missing outcome data** | **Measurement of the outcome** | **Selection of the reported result** | **Overall Bias** |
| --- | --- | --- | --- | --- | --- | --- |
| Ahmadzadeh et al (2018) | Low risk | Low risk | Low risk | Low risk | Low risk | Low risk |
| Neil Mori et al (2015) | Low risk | Low risk | Low risk | Low risk | Low risk | Low risk |
| Conor W Kelly et al (2019) | Low risk | Low risk | Low risk | Low risk | Low risk | Low risk |
| Farah et al (2015) | Low risk | Low risk | Low risk | Low risk | Low risk | Low risk |

Table S5: Cochrane Risk of Bias tool 2.0 for randomized controlled trials (RCTs).
